# The genome sequence of the Large Scabious Mining Bee, *Andrena hattorfiana* (Fabricius, 1775)

**DOI:** 10.12688/wellcomeopenres.19438.1

**Published:** 2023-05-18

**Authors:** Steven Falk, Kar-Tong Tan, University of Oxford and Wytham Woods Genome Acquisition Lab, Darwin Tree of Life Barcoding collective, Wellcome Sanger Institute Tree of Life programme, Wellcome Sanger Institute Scientific Operations: DNA Pipelines collective, Tree of Life Core Informatics collective, Darwin Tree of Life Consortium

**Affiliations:** 1 Independent Researcher, Kenilworth, England, UK; 2 Harvard University, Cambridge, Massachusetts, USA

**Keywords:** Andrena hattorfiana, Large Scabious Mining Bee, genome sequence, chromosomal; Hymenoptera

## Abstract

We present a genome assembly from an individual female *Andrena hattorfiana* (the Large Scabious Mining Bee; Arthropoda; Insecta; Hymenoptera; Andrenidae). The genome sequence is 428.5 megabases in span. Most of the assembly is scaffolded into seven chromosomal pseudomolecules. The mitochondrial genome has also been assembled and is 22.7 kilobases in length. Gene annotation of this assembly on Ensembl identified 11,349 protein coding genes.

## Species taxonomy

Eukaryota; Metazoa; Ecdysozoa; Arthropoda; Hexapoda; Insecta; Pterygota; Neoptera; Endopterygota; Hymenoptera; Apocrita; Aculeata; Apoidea; Andrenidae; Andreninae; *Andrena*; *Andrena hattorfiana* (Fabricius, 1775) (NCBI:txid1126402).

## Background

*Andrena hattorfiana* is a species of mining bee commonly found throughout Europe, from the south of the Scandinavian countries to north Africa, and eastwards to the Caucasus, but its population has declined in Europe and the UK due to habitat loss ( Dimond, 2015). In contrast to the common European bee that lives collectively in a colony, *A. hattorfiana* is solitary and constructs nests in the ground, where it lays its eggs and provisions them with a mixture of pollen and nectar ( Larsson & Franzén, 2007; Dimond, 2015).

*Andrena hattorfiana* is oligolectic, feeding on pollen from a single family or genus of flowering plants ( Cane & Sipes, 2006; Larsson & Franzén, 2007). Specifically, it feeds on pollen from the flowers of *Knautia arvensis* and *Scabiosa columbaria* ( Jefferson, 2022; Reemer *et al.*, 2012; Varga *et al.*, 2022). Given its limited diet, *A. hattorfiana* is endangered due to a number of factors: pollen competition, not enough variability in its habitat, and because of the dearth of traditionally managed meadows ( Carvell *et al.*, 2006; Falk, 1991; Larsson & Franzén, 2007; Varga *et al.*, 2022) and the effects of climate, which exacerbates habitat loss.

The generation of a reference genome sequence for *A. hattorfiana* is likely to aid in the preservation efforts for this vulnerable species, as well as to help in the understanding of the broader biology of this species. For instance, the reference genome would help in subsequent assessment of whether there is sufficient genetic diversity in the population for it to adapt to changing conditions, if deleterious genes are becoming fixed in the population ( MacDonald, 2021), and for identifying genetic elements that uniquely characterise *A. hattorfiana* versus other species of bees.

## Genome sequence report

**Figure 1.**
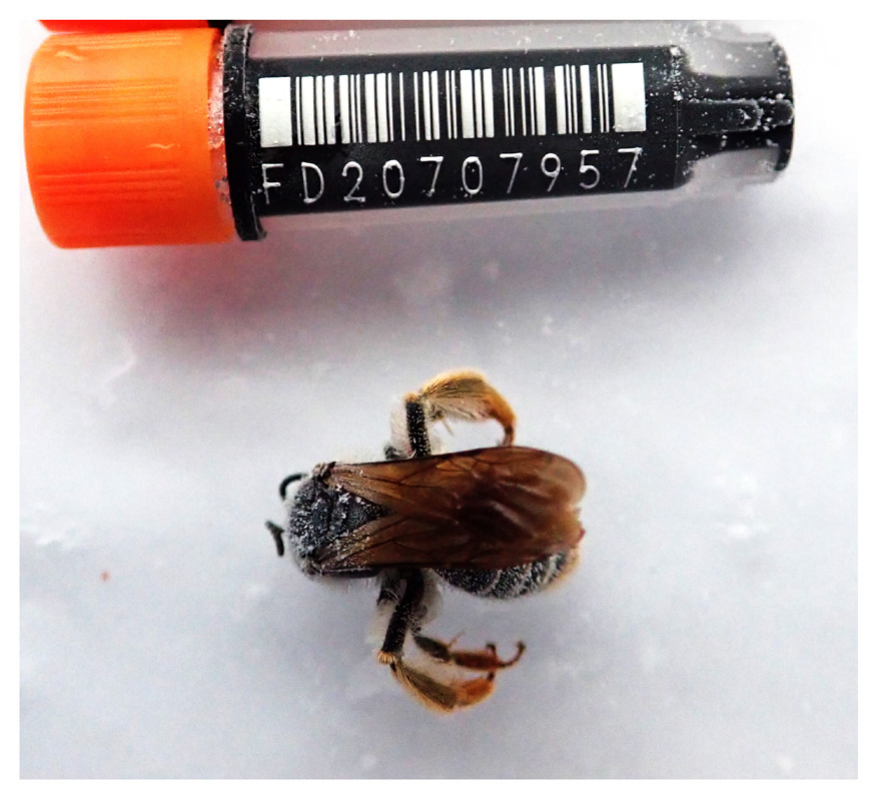
Photograph of the *Andrena hattorfiana* (iyAndHatt1) specimen used for genome sequencing.

The genome was sequenced from one female *Andrena hattorfiana* specimen ( Figure 1) collected from Wytham Woods, Oxfordshire (biological vice-county: Berkshire), UK (latitude 51.77, longitude –1.33). A total of 49-fold coverage in Pacific Biosciences single-molecule HiFi long reads was generated. Primary assembly contigs were scaffolded with chromosome conformation Hi-C data. Manual assembly curation corrected 134 missing joins or mis-joins, reducing the scaffold number by 49.6%, and increasing the scaffold N50 by 196.76%.

**Figure 2.**
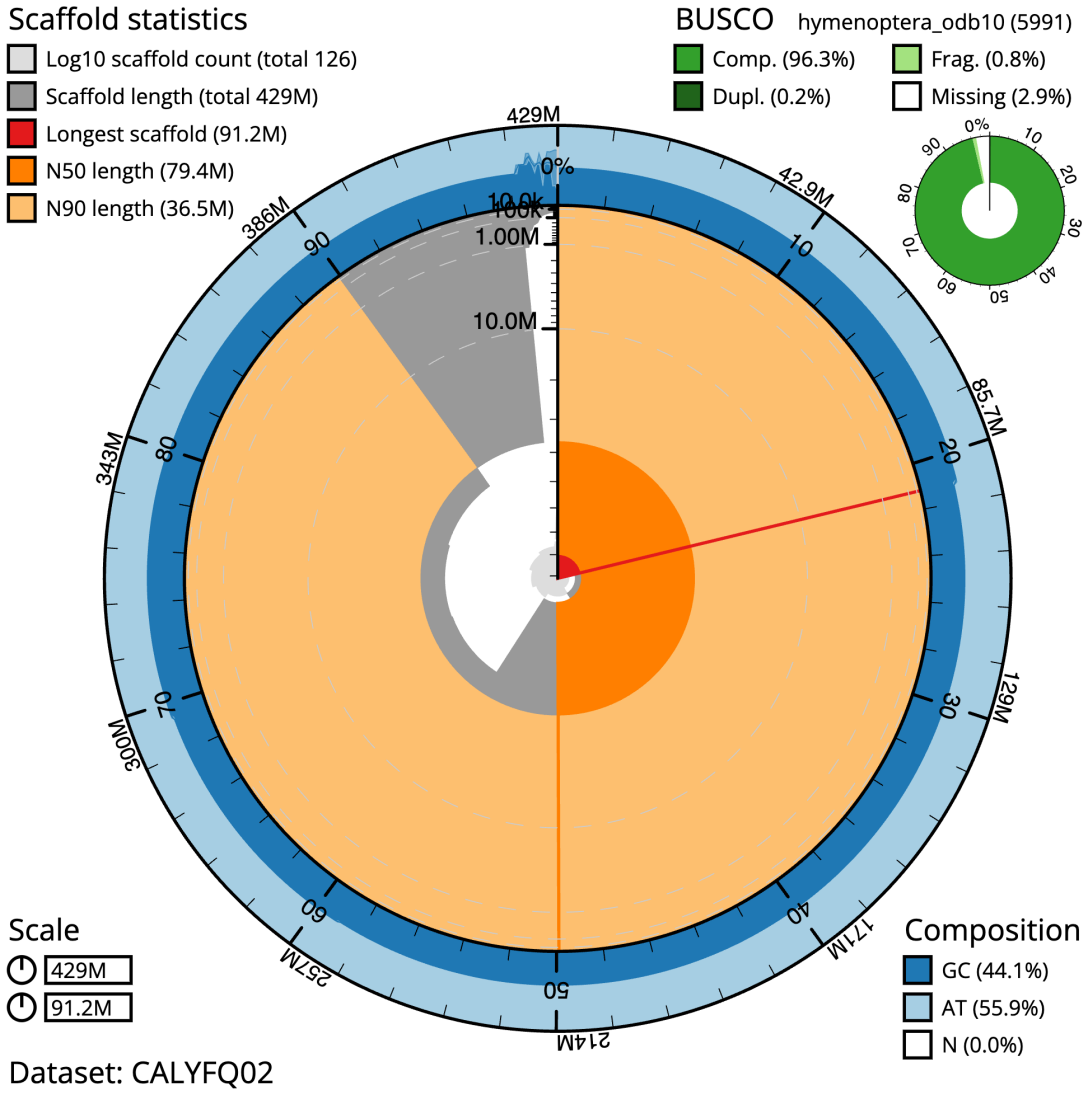
Genome assembly of *Andrena hattorfiana*, iyAndHatt1.2: metrics. The BlobToolKit Snailplot shows N50 metrics and BUSCO gene completeness. The main plot is divided into 1,000 size-ordered bins around the circumference with each bin representing 0.1% of the 428,532,965 bp assembly. The distribution of scaffold lengths is shown in dark grey with the plot radius scaled to the longest sequence present in the assembly (91,187,844 bp, shown in red). Orange and pale-orange arcs show the N50 and N90 scaffold lengths (79,350,654 and 36,459,576 bp), respectively. The pale grey spiral shows the cumulative scaffold count on a log scale with white scale lines showing successive orders of magnitude. The blue and pale-blue area around the outside of the plot shows the distribution of GC, AT and N percentages in the same bins as the inner plot. A summary of complete, fragmented, duplicated and missing BUSCO genes in the hymenoptera_odb10 set is shown in the top right. An interactive version of this figure is available at https://blobtoolkit.genomehubs.org/view/iyAndHatt1.2/dataset/CALYFQ02/snail.

The final assembly has a total length of 428.5 Mb in 125 sequence scaffolds with a scaffold N50 of 79.4 Mb ( Table 1). Most (98.36%) of the assembly sequence was assigned to seven chromosomal-level scaffolds. Chromosome-scale scaffolds confirmed by the Hi-C data are named in order of size ( Figure 2– Figure 5; Table 2). The scaffold order and orientation are uncertain in the following regions: chromosome 1 (61.37–77.87 Mb), chromosome 3 (19.30–20.39 Mb and 29.90–33.14 Mb), and chromosome 5 (20.86–26.68 Mb). The mitochondrial genome was also assembled and can be found as a contig within the multifasta file of the genome submission.

**Table 1.** Genome data for *Andrena hattorfiana*, iyAndHatt1.2.

Project accession data		
Assembly identifier	iyAndHatt1.2	
Species	*Andrena hattorfiana*	
Specimen	iyAndHatt1	
NCBI taxonomy ID	1126402	
BioProject	PRJEB52207	
BioSample ID	SAMEA7746468	
Isolate information	iyAndHatt1, female	
Assembly metrics *		*Benchmark*
Consensus quality (QV)	64.5	*≥ 50*
*k*-mer completeness	100%	*≥ 95%*
BUSCO **	C:96.3%[S:96.1%,D:0.2%], F:0.8%,M:2.9%,n:5,991	*C ≥ 95%*
Percentage of assembly mapped to chromosomes	98.36%	*≥ 95%*
Sex chromosomes	Not applicable	*localised homologous pairs*
Organelles	Mitochondrial genome assembled	*complete single alleles*
Raw data accessions		
PacificBiosciences SEQUEL II	ERR9630942	
Hi-C Illumina	ERR9580473	
PolyA RNA-Seq Illumina	ERR10123693	
Genome assembly		
Assembly accession	GCA_944738655.2	
*Accession of alternate haplotype*	GCA_944738785.2	
Span (Mb)	428.5	
Number of contigs	310	
Contig N50 length (Mb)	10.2	
Number of scaffolds	125	
Scaffold N50 length (Mb)	79.4	
Longest scaffold (Mb)	91.2	
Genome annotation		
Number of protein-coding genes	11,349	
Number of non-coding genes	4,338	
Number of gene transcripts	26,897	

* Assembly metric benchmarks are adapted from column VGP-2020 of “Table 1: Proposed standards and metrics for defining genome assembly quality” from ( Rhie *et al.*, 2021). ** BUSCO scores based on the hymenoptera_odb10 BUSCO set using v5.3.2. C = complete [S = single copy, D = duplicated], F = fragmented, M = missing, n = number of orthologues in comparison. A full set of BUSCO scores is available at https://blobtoolkit.genomehubs.org/view/iyAndHatt1.2/dataset/CALYFQ02/busco.

**Figure 3.**
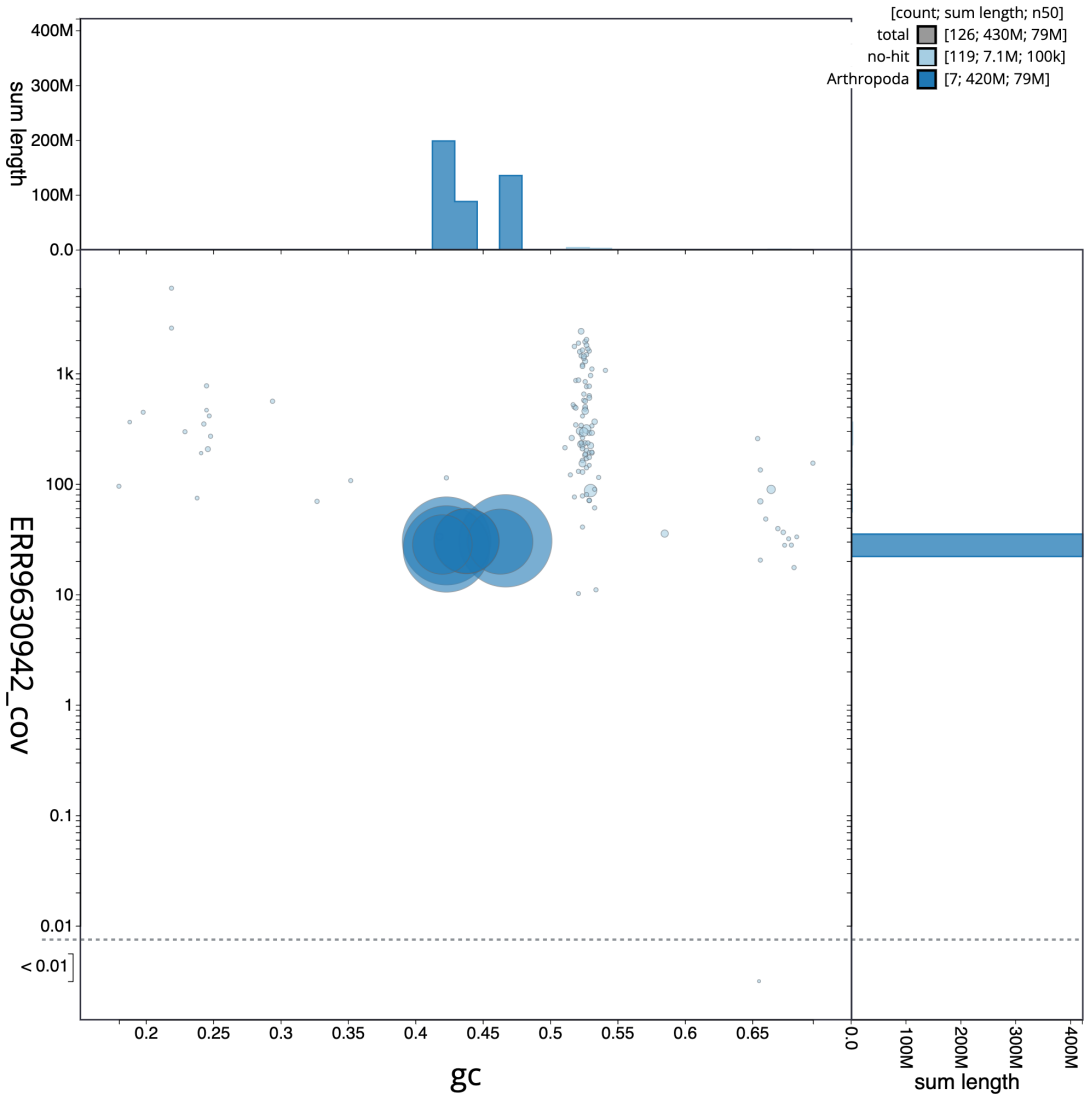
Genome assembly of *Andrena hattorfiana*, iyAndHatt1.2: GC coverage. BlobToolKit GC-coverage plot. Scaffolds are coloured by phylum. Circles are sized in proportion to scaffold length. Histograms show the distribution of scaffold length sum along each axis. An interactive version of this figure is available at https://blobtoolkit.genomehubs.org/view/iyAndHatt1.2/dataset/CALYFQ02/blob.

**Figure 4.**
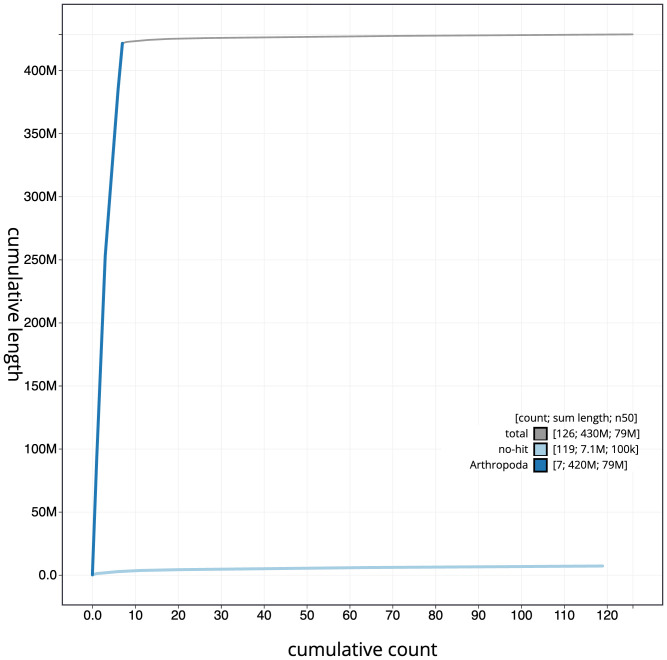
Genome assembly of *Andrena hattorfiana*, iyAndHatt1.2: cumulative sequence. BlobToolKit cumulative sequence plot. The grey line shows cumulative length for all scaffolds. Coloured lines show cumulative lengths of scaffolds assigned to each phylum using the buscogenes taxrule. An interactive version of this figure is available at https://blobtoolkit.genomehubs.org/view/iyAndHatt1.2/dataset/CALYFQ02/cumulative.

**Figure 5.**
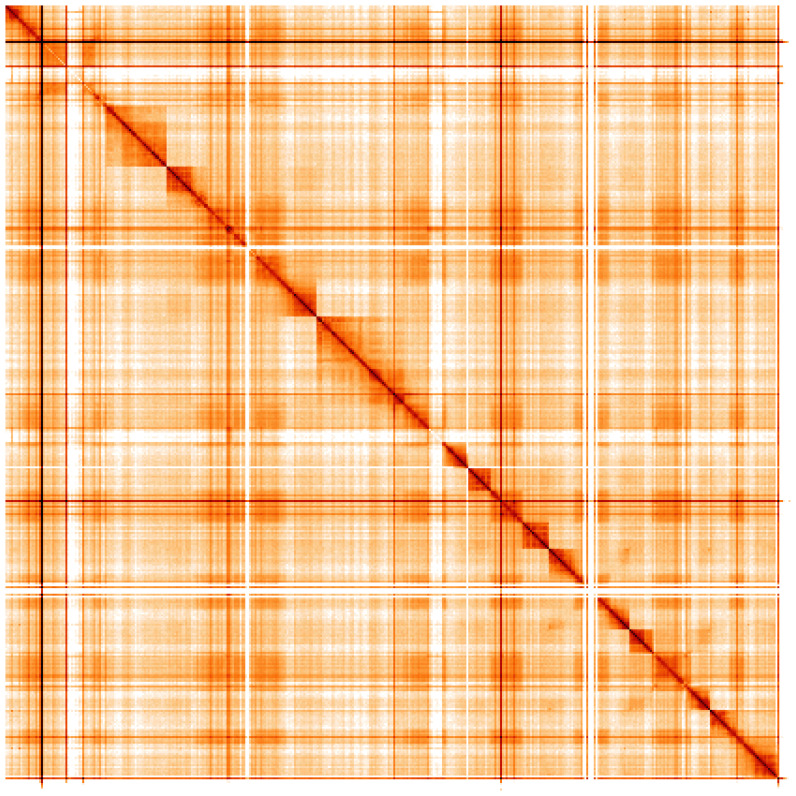
Genome assembly of *Andrena hattorfiana*, iyAndHatt1.2: Hi-C contact map. Hi-C contact map of the iyAndHatt1.2 assembly, visualised using HiGlass. Chromosomes are shown in order of size from left to right and top to bottom. An interactive version of this figure may be viewed at https://genome-note-higlass.tol.sanger.ac.uk/l/?d=NVN2MuBATkat5Wk8tNuFqA.

**Table 2.** Chromosomal pseudomolecules in the genome assembly of *Andrena hattorfiana*, iyAndHatt1.

INSDC accession	Chromosome	Size (Mb)	GC%
OX122889.2	1	91.19	44.5
OX122890.1	2	82.47	42.3
OX122891.2	3	79.35	44
OX122892.1	4	44.41	43.8
OX122893.2	5	44,10	46
OX122894.2	6	43.51	43.5
OX122895.1	7	36.46	42
OX122896.1	MT	0.02	22.9

The estimated Quality Value (QV) of the final assembly is 64.5 with *k*-mer completeness of 100%, and the assembly has a BUSCO v5.3.2 completeness of 96.3% (single = 96.1%, duplicated = 0.2%), using the hymenoptera_odb10 reference set ( *n* = 5,991).

Metadata for specimens, spectral estimates, sequencing runs, contaminants and pre-curation assembly statistics can be found at https://links.tol.sanger.ac.uk/species/1126402.

## Genome annotation report

The *Andrena hattorfiana* genome assembly (GCA_944738655.1, iyAndHatt1.1) was annotated using the Ensembl rapid annotation pipeline ( Table 1; https://rapid.ensembl.org/Andrena_hattorfiana_GCA_944738655.1/). The resulting annotation includes 26,897 transcribed mRNAs from 11,349 protein-coding and 4,338 non-coding genes.

## Methods

### Sample acquisition and nucleic acid extraction

A female *Andrena hattorfiana* specimen (iyAndHatt1) was collected from Wytham Woods, Oxfordshire (biological vice-county: Berkshire), UK (latitude 51.77, longitude –1.33) on 4 August 2020. The specimen was taken from woodland habitat by Steven Falk (independent researcher) by netting. The specimen was identified by Steven Falk and preserved on dry ice.

DNA was extracted at the Tree of Life laboratory, Wellcome Sanger Institute (WSI). The iyAndHatt1 sample was weighed and dissected on dry ice with tissue set aside for Hi-C sequencing. Thorax tissue was disrupted using a Nippi Powermasher fitted with a BioMasher pestle. High molecular weight (HMW) DNA was extracted using the Qiagen MagAttract HMW DNA extraction kit. HMW DNA was sheared into an average fragment size of 12–20 kb in a Megaruptor 3 system with speed setting 30. Sheared DNA was purified by solid-phase reversible immobilisation using AMPure PB beads with a 1.8X ratio of beads to sample to remove the shorter fragments and concentrate the DNA sample. The concentration of the sheared and purified DNA was assessed using a Nanodrop spectrophotometer and Qubit Fluorometer and Qubit dsDNA High Sensitivity Assay kit. Fragment size distribution was evaluated by running the sample on the FemtoPulse system.

RNA was extracted from abdomen tissue of iyAndHatt1 in the Tree of Life Laboratory at the WSI using TRIzol, according to the manufacturer’s instructions. RNA was then eluted in 50 μl RNAse-free water and its concentration assessed using a Nanodrop spectrophotometer and Qubit Fluorometer using the Qubit RNA Broad-Range (BR) Assay kit. Analysis of the integrity of the RNA was done using Agilent RNA 6000 Pico Kit and Eukaryotic Total RNA assay.

### Sequencing

Pacific Biosciences HiFi circular consensus DNA sequencing libraries were constructed according to the manufacturers’ instructions. Poly(A) RNA-Seq libraries were constructed using the NEB Ultra II RNA Library Prep kit. DNA and RNA sequencing was performed by the Scientific Operations core at the WSI on Pacific Biosciences SEQUEL II (HiFi) and Illumina NovaSeq 6000 (RNA-Seq) instruments. Hi-C data were also generated from head tissue of iyAndHatt1 using the Arima v2 kit and sequenced on the Illumina NovaSeq 6000 instrument.

### Genome assembly, curation and evaluation

Assembly was carried out with Hifiasm ( Cheng *et al.*, 2021) and haplotypic duplication was identified and removed with purge_dups ( Guan *et al.*, 2020). The assembly was scaffolded with Hi-C data ( Rao *et al.*, 2014) using YaHS ( Zhou *et al.*, 2023). The assembly was checked for contamination as described previously ( Howe *et al.*, 2021). Manual curation was performed using HiGlass ( Kerpedjiev *et al.*, 2018) and Pretext ( Harry, 2022). The mitochondrial genome was assembled using MitoHiFi ( Uliano-Silva *et al.*, 2022), which runs MitoFinder ( Allio *et al.*, 2020) or MITOS ( Bernt *et al.*, 2013) and uses these annotations to select the final mitochondrial contig and to ensure the general quality of the sequence.

A Hi-C map for the final assembly was produced using bwa-mem2 ( Vasimuddin *et al.*, 2019) in the Cooler file format ( Abdennur & Mirny, 2020). To assess the assembly metrics, the *k*-mer completeness and QV consensus quality values were calculated in Merqury ( Rhie *et al.*, 2020). This work was done using Nextflow ( Di Tommaso *et al.*, 2017) DSL2 pipelines “sanger-tol/readmapping” ( Surana *et al.*, 2023a) and “sanger-tol/genomenote” ( Surana *et al.*, 2023b). The genome was analysed within the BlobToolKit environment ( Challis *et al.*, 2020) and BUSCO scores ( Manni *et al.*, 2021; Simão *et al.*, 2015) were calculated.

Table 3 contains a list of relevant software tool versions and sources.

**Table 3.** Software tools: versions and sources.

Software tool	Version	Source
BlobToolKit	4.1.3	https://github.com/blobtoolkit/blobtoolkit
BUSCO	5.3.2	https://gitlab.com/ezlab/busco
Hifiasm	0.16.1-r375	https://github.com/chhylp123/hifiasm
HiGlass	1.11.6	https://github.com/higlass/higlass
Merqury	MerquryFK	https://github.com/thegenemyers/MERQURY.FK
MitoHiFi	2	https://github.com/marcelauliano/MitoHiFi
PretextView	0.2	https://github.com/wtsi-hpag/PretextView
purge_dups	1.2.3	https://github.com/dfguan/purge_dups
sanger-tol/genomenote	v1.0	https://github.com/sanger-tol/genomenote
sanger-tol/readmapping	1.1.0	https://github.com/sanger-tol/readmapping/tree/1.1.0
YaHS	yahs-1.1.91eebc2	https://github.com/c-zhou/yahs

### Genome annotation

The Ensembl gene annotation system ( Aken *et al.*, 2016) was used to generate annotation for the *Andrena hattorfiana* assembly (GCA_944738655.1). Annotation was created primarily through alignment of transcriptomic data to the genome, with gap filling via protein-to-genome alignments of a select set of proteins from UniProt ( UniProt Consortium, 2019).

### Ethics and compliance issues

The materials that have contributed to this genome note have been supplied by a Darwin Tree of Life Partner. The submission of materials by a Darwin Tree of Life Partner is subject to the Darwin Tree of Life Project Sampling Code of Practice. By agreeing with and signing up to the Sampling Code of Practice, the Darwin Tree of Life Partner agrees they will meet the legal and ethical requirements and standards set out within this document in respect of all samples acquired for, and supplied to, the Darwin Tree of Life Project. Each transfer of samples is further undertaken according to a Research Collaboration Agreement or Material Transfer Agreement entered into by the Darwin Tree of Life Partner, Genome Research Limited (operating as the Wellcome Sanger Institute), and in some circumstances other Darwin Tree of Life collaborators.

## Data Availability

European Nucleotide Archive: *Andrena hattorfiana* (large scabious mining bee). Accession number PRJEB52207; https://identifiers.org/ena.embl/PRJEB52207 ( Wellcome Sanger Institute, 2022). The genome sequence is released openly for reuse. The *Andrena hattorfiana* genome sequencing initiative is part of the Darwin Tree of Life (DToL) project. All raw sequence data and the assembly have been deposited in INSDC databases. Raw data and assembly accession identifiers are reported in Table 1.

## Author information

Members of the University of Oxford and Wytham Woods Genome Acquisition Lab are listed here: https://doi.org/10.5281/zenodo.4789928.

Members of the Darwin Tree of Life Barcoding collective are listed here: https://doi.org/10.5281/zenodo.4893703.

Members of the Wellcome Sanger Institute Tree of Life programme are listed here: https://doi.org/10.5281/zenodo.4783585.

Members of Wellcome Sanger Institute Scientific Operations: DNA Pipelines collective are listed here: https://doi.org/10.5281/zenodo.4790455.

Members of the Tree of Life Core Informatics collective are listed here: https://doi.org/10.5281/zenodo.5013541.

Members of the Darwin Tree of Life Consortium are listed here: https://doi.org/10.5281/zenodo.4783558.
