# Supplementary material for: The genome sequence of the Large Scabious Mining Bee, Andrena hattorfiana (Fabricius, 1775)
Source: Wellcome Open Res. 2023 May 18;8:224. [Version 1] doi: 10.12688/wellcomeopenres.19438.1 (PMC10511852; doi:10.12688/wellcomeopenres.19438.1)
Supplement: Peer review history

# Reviewer report: Reviewer response for version 1

**DOI:** 10.21956/wellcomeopenres.21534.r67330

**Published:** 2023-10-03

**Authors:** Miriam Richards

**Affiliations:** 1 Department of Biological Sciences, Brock University, Saint Catharines, Ontario, Canada

Falk and Tan The genome sequence of the Large Scabious Mining Bee, *Andrena hattorfiana*

 This paper provides a chromosome-level genome sequence for the solitary andrenid, *A. hattorfiana*, a common species which nevertheless may be declining across Europe.  The genome was sequenced from a single female specimen collected from the UK.  The sequencing quality is high, based on comparison to several benchmarks.  An initial estimate of genome size (428.5 Mb) places it at the high end for bees, with seven chromosomal-level scaffolds, identifying 11349 protein-coding genes, 4338 non-coding genes, and ~27,000 different transcripts. The data were filtered to ensure that the genome sequences were not from parasites or symbionts.  Having a genome sequence is likely to be useful in conservation efforts, noting that a reference genome will be useful in future conservation genetic and taxonomic studies. I agree with this statement, and I am happy to see this new bee genome.  I suspect that as bee genomes accumulated, many kinds of useful comparative studies will be enabled in other fields as well, including bioinformatics and molecular evolution.

 A minor concern with the paper is that there is no mention of voucher specimens, in case future studies raise queries about the identify of the sequenced specimen.  I suppose a DNA barcode, inherent in the genome, could be enough to address this issue.  A second minor concern is that the bioinformatics pipelines are described, but it would be useful to provide details of options and switches that may have been used with each piece of software.  I'm aware that few papers provide sufficient bioinformatics detail to easily replicate the methods! I was pleased to see Table 3, which simplifies the tracking down of software tools.

Are sufficient details of methods and materials provided to allow replication by others?

Partly

Is the rationale for creating the dataset(s) clearly described?

Yes

Are the datasets clearly presented in a useable and accessible format?

Yes

Are the protocols appropriate and is the work technically sound?

Yes

Reviewer Expertise:

Bee ecology, behaviour, evolution, bee genomes

I confirm that I have read this submission and believe that I have an appropriate level of expertise to confirm that it is of an acceptable scientific standard.

# Reviewer report: Reviewer response for version 1

**DOI:** 10.21956/wellcomeopenres.21534.r67328

**Published:** 2023-10-03

**Authors:** Lucio Navarro-Escalante

**Affiliations:** 1 Department of Molecular Biosciences, The University of Texas at Austin, Austin, Texas, USA

This report presents the full genome sequences of the large scabious mining bee, *Andrena* *hattorfiana*, an endangered Hymenoptera species. This is a high quality genome assembly at chromosome-scale level performed using state-of-the-art DNA sequencing technologies. The method section describes sufficiently each one of the methodological steps followed. The sequencing, assembly and annotation data are clearly described in the report and they are available from repositories for the scientific community. Despite the few comments I have bellow, I support the indexing of this report and its data.

**Comments**:

**Background**:

1. Define clearly the species name for the “common European bee”.
2. Consider including a small paragraph or some lines highlighting better the relevance of the large scabious mining bee. To my understanding the bee is listed provisionally within the UK “Nationally Rare Species”.

**Results**:

1. I would suggest to delimit and name the putative chromosome boundaries in the Hi-C contact map in Figure 5.

**Methods**:

1. Describe in more detail how the specimen was taxonomically identified. Did you used morphological or molecular characters (e.g. COI sequences)?
2. Please, add a brief description of the overall method or name the tool used for checking contamination in the genome assembly.
3. There should be an explanation of why only the abdomen was used for RNA isolation and sequencing.

Are sufficient details of methods and materials provided to allow replication by others?

Yes

Is the rationale for creating the dataset(s) clearly described?

Yes

Are the datasets clearly presented in a useable and accessible format?

Yes

Are the protocols appropriate and is the work technically sound?

Yes

Reviewer Expertise:

Arthopod genomics, insect-microbial symbiosis, insect-plant interactions.

I confirm that I have read this submission and believe that I have an appropriate level of expertise to confirm that it is of an acceptable scientific standard.

# Reviewer report: Reviewer response for version 1

**DOI:** 10.21956/wellcomeopenres.21534.r67329

**Published:** 2023-09-26

**Authors:** Denilce Lopes

**Affiliations:** 1 Departamento de Biologia Geral, Universidade Federal de Vicosa, Viçosa, State of Minas Gerais, Brazil

This report accurately describes the genome assembly process of Andrena, which seems of good quality with good N50 value and BUSCO score. My concern with this manuscript and the other Data Notes from this Consortium is that, except for the Background item, the text is practically the same, changing only the name of the species and the sequencing data. I suggest reviewing this for future publications. In particular for bees, few complete genomes have been sequenced and the data will be useful in preservation efforts and other behavioral studies on these insects.

Are sufficient details of methods and materials provided to allow replication by others?

Yes

Is the rationale for creating the dataset(s) clearly described?

Yes

Are the datasets clearly presented in a useable and accessible format?

Yes

Are the protocols appropriate and is the work technically sound?

Yes

Reviewer Expertise:

Cytogenomic of Hymenoptera

I confirm that I have read this submission and believe that I have an appropriate level of expertise to confirm that it is of an acceptable scientific standard.

# Reviewer report: Reviewer response for version 1

**DOI:** 10.21956/wellcomeopenres.21534.r65567

**Published:** 2023-09-20

**Authors:** Mark Harrison

**Affiliations:** 1 Institute for Evolution and Biodiversity, University of Munster, Munster, Germany

The authors present the genome assembly of the solitary mining bee, *Andrena hattorfiana*, within the Darwin tree of life project. The DToL is an extremely important and highly professional project. Also here, a high quality genome assembly is presented that has been sequenced, assembled and annotated with state-of-the-art methods. I support the publication of this manuscript with just a couple of notes below.

**Introduction, first paragraph:** What is the common European bee? Are the authors referring to the honey bee, *Apis mellifera*? This should be more specific. Also the wording could suggest that most bees are eusocial, which they're not. Maybe change to something like, unlike the eusocial honey bee, *Apis mellifera, A hatt*. is solitary....

**Genome sequence report:**

 How much Hi-C and HiFi data were generated in total?

**Genome annotation report:**

 What is the completeness of the annotated genes? A BUSCO analysis could be helpful

**Methods:**

 Which UniProt protein dataset was used? It is quite vague.

Are sufficient details of methods and materials provided to allow replication by others?

Yes

Is the rationale for creating the dataset(s) clearly described?

Yes

Are the datasets clearly presented in a useable and accessible format?

Yes

Are the protocols appropriate and is the work technically sound?

Yes

Reviewer Expertise:

Insect genomics, comparative genomics, evolution of complex traits

I confirm that I have read this submission and believe that I have an appropriate level of expertise to confirm that it is of an acceptable scientific standard.

# Reviewer report: Reviewer response for version 1

**DOI:** 10.21956/wellcomeopenres.21534.r65569

**Published:** 2023-08-22

**Authors:** Michael Orr

**Affiliations:** 1 Entomologie, Staatliches Museum fur Naturkunde Stuttgart, Stuttgart, Baden-Württemberg, Germany

This paper describes the genome generation of a species of vulnerable bee, and so could be useful for future conservation work, as the authors aptly note. I am not myself an expert of genome quality assessment/etc. but Darwin Tree of Life has already sequenced many bees and numerous Andrena successfully so their practices have assumedly been very well refined by now. Generally, the parameters I'm familiar with seem reasonable overall and the openness and completeness of the methods and data sourcing look good to me. The genome size is also pretty close to the other Andrena they’ve sequenced (with one exception I noticed being A. dorsata with <3mb but that’d be more a potential issue with that assembly?), which bodes well. Overall, I appreciate the efforts of Darwin Tree of Life and am glad for the many new questions that can be approached with these genomes.

 Overall, I think that this can be approved and that just some small changes and information added would suffice. My comments are generally rather simple and should be easy to incorporate (although without line numbers or a way to upload tracked changes files it's a little cumbersome...).

 Specific comments are given below:

 Background:

- First paragraph ascribes their conservation worries to habitat loss but the second paragraph notes specialization may also play a role, maybe best to explain the underlying cause in one place and using the more complete second listing of potential factors. The first mention could just be that it's listed as vulnerable by the IUCN (which a citation thereof). 
- "the common European bee" in the first paragraph is very vague. Please say honey bee explicitly and list *Apis mellifera* in parentheses. 
- Maybe not necessary, but another interesting use of the genome that could be mentioned in the third paragraph is the relevance for understanding the genomic toolkits of floral specialists in general.

Methods

- A bit more should be stated under sample acquisition. ID was in the field, correct? Or was it using a leg later sequenced for COI, or pulling COI from the genome sequence? Are there vouchers that were collected at the same site? Where are they deposited if so?
- Third paragraph on RNA: shouldn’t a whole body have been used for RNA? I guess maybe there are limits on collecting these vulnerable species though…? This could be stated somewhere in the methods explicitly as justification. Also, was the gut removed? We usually did this to reduce the number of nontarget reads but I think it’s now less necessary as long read tech matures

Are sufficient details of methods and materials provided to allow replication by others?

Partly

Is the rationale for creating the dataset(s) clearly described?

Yes

Are the datasets clearly presented in a useable and accessible format?

Yes

Are the protocols appropriate and is the work technically sound?

Yes

Reviewer Expertise:

Bee biodiversity, conservation, and systematics.

I confirm that I have read this submission and believe that I have an appropriate level of expertise to confirm that it is of an acceptable scientific standard.
